# Allostatic Load and Stress Physiology in European Seabass (*Dicentrarchus labrax* L.) and Gilthead Seabream (*Sparus aurata* L.)

**DOI:** 10.3389/fendo.2018.00451

**Published:** 2018-08-13

**Authors:** Athanasios Samaras, Carlos Espírito Santo, Nikos Papandroulakis, Nikolaos Mitrizakis, Michail Pavlidis, Erik Höglund, Thamar N. M. Pelgrim, Jan Zethof, F. A. Tom Spanings, Marco A. Vindas, Lars O. E. Ebbesson, Gert Flik, Marnix Gorissen

**Affiliations:** ^1^AquaLabs, Institute of Marine Biology, Biotechnology and Aquaculture, Hellenic Centre for Marine Research, Heraklion, Greece; ^2^Department of Biology, University of Crete, Heraklion, Greece; ^3^Department of Animal Ecology and Physiology, Institute for Water and Wetland Research, Radboud University, Nijmegen, Netherlands; ^4^Norwegian Institute for Water Research (NIVA), Oslo, Norway; ^5^Section for Aquaculture, National Institute of Aquatic Resources, Technical University of Denmark, Hirtshals, Denmark; ^6^Uni Research Environment, Bergen, Norway

**Keywords:** allostasis, aquaculture, cortisol, CRF, repeated stress, serotonin

## Abstract

The present study aimed to compare effects of increasing chronic stress load on the stress response of European seabass (*Dicentrarchus labrax*) and gilthead seabream (*Sparus aurata*) to identify neuroendocrine functions that regulate this response. Fish were left undisturbed (controls) or exposed to three levels of chronic stress for 3 weeks and then subjected to an acute stress test (ACT). Chronic stress impeded growth and decreased feed consumption in seabass, not in seabream. In seabass basal cortisol levels are high and increase with stress load; the response to a subsequent ACT decreases with increasing (earlier) load. Basal cortisol levels in seabream increase with the stress load, whereas the ACT induced a similar response in all groups. In seabass and seabream plasma α-MSH levels and brain stem serotonergic activity and turnover were similar and not affected by chronic stress. Species-specific molecular neuro-regional differences were seen. *In-situ* hybridization analysis of the early immediate gene *cfos* in the preoptic area showed ACT-activation in seabream; in seabass the expression level was not affected by ACT and seems constitutively high. In seabream, expression levels of telencephalic *crf, crfbp, gr1*, and *mr* were downregulated; the seabass hypothalamic preoptic area showed increased expression of *crf* and *gr1*, and decreased expression of *mr*, and this increased the *gr1*/*mr* ratio considerably. We substantiate species-specific physiological differences to stress coping between seabream and seabass at an endocrine and neuroendocrine molecular level. Seabass appear less resilient to stress, which we conclude from high basal activities of stress-related parameters and poor, or absent, responses to ACT. This comparative study reveals important aquaculture, husbandry, and welfare implications for the rearing of these species.

## Introduction

The concept of allostasis, which states that animals “achieve constancy through change” [adjusting set points of regulatory loops to prevailing needs; (1, 2)] is gaining popularity in fish stress physiology. Allostasis involves synthesis of prior knowledge with predicted current needs and resetting of one or more physiological set points accordingly. A successful stress response involves the reorganization of the organism's energy budget, their immune system, as well as neural and endocrine mechanisms to successfully cope with a given stressor. The stress response then results in a timely return to pre-stress conditions, and restoration of homeostasis so-called eustress (3). If the response fails, or is inadequate, allostatic overload will occur. This is usually seen under chronic stress conditions when individuals are no longer able to successfully cope with continued stress challenge (4). The term “allostatic load” is used to describe the capacity of an organism to cope with a certain challenge by acclimating its behavior and physiology. Stress responses are meant to be compensatory and adaptive, to allow the animal to overcome the threat; when the animal succeeds in this we refer to stress as eustress. However, when an animal is facing an intense or chronic stress, the stress response might lose its adaptive significance, become dysfunctional and ultimately result in adverse effects such as inhibition of growth, failure to reproduce, and impeded resistance to pathogens. This condition is called distress (3, 5–7).

The stress response in fish (in fact in any vertebrate) is initiated by activating the hypothalamic–sympathetic axis followed by the activation of the hypothalamus–pituitary gland–interrenal gland (HPI) axis. The former results in the release of adrenaline and noradrenaline to quickly induce hyperglycemia and fuel fight or flight (3, 8). However, due to the rapid release and clearing of catecholamines from the circulation [seconds to minutes; (9)] it is difficult to obtain accurate data on the resting levels of adrenaline and noradrenaline, and for that reason these parameters are not commonly assayed. The endocrine stress steroid axis (HPI-axis) will subsequently produce (hyperglycemic) cortisol to guarantee energy for coping with the new conditions and counteract changes in energy budgeting induced by the stressor. Indeed, corticotrophin-releasing factor (CRF) is secreted from the preoptic area [POA; (10–13)]. The axons of CRF-producing cells project directly to *pars distalis* ACTH cells (12). CRF is released there and will then bind the CRF-receptors (CRF_1_R) located on the ACTH cells (12). This process is believed to be modulated by CRF-binding-protein (CRF-BP), which binds CRF and therefore reduces its bioavailability (12, 14, 15). Hypothalamic CRF neurons also project to the pituitary *pars intermedia* and induce release of α-melanophore-stimulating hormone (α-MSH) (16, 17); in particular, increased constitutive release under conditions of chronic stress (18, 19) may act as corticotrope, lipolytic or anorexigenic signal (3).

ACTH acts via a specific melanocortin receptor type 2 (MC2R), expressed exclusively on interrenal cells in the head kidney of fish (16, 20); this receptor acts as a dimer and is associated with four melanocortin receptor associated proteins [MRAPs; (21–23)]. MC2R activates pathways that result in synthesis of cortisol from cholesterol and subsequent secretion to the bloodstream (24). The mechanisms regulating its expression are not yet fully described, but in seabass it seems that exogenous cortisol administration can exert negative feedback on *mc2r* gene expression (20). Cortisol, the single steroid produced by interrenal cells in fish, signals in target tissues *via* either a mineralocorticoid or several glucocorticoid receptors (MR and GRs, respectively). Once cortisol is bound, these transcription factors bind specific DNA sequences (GR- and MR-responsive elements) in target-gene promoters and control mineralocorticoid and glucocorticoid activities as required to cope with imposed challenges (8, 25, 26).

The aim of the present study was to study the neuroendocrine regulation of European seabass (*Dicentrarchus labrax* L.) and gilthead seabream (*Sparus aurata* L.) upon exposure to different intensities and types of chronic stress. These species constitute the largest portion (approximately 90–95%) of the Mediterranean aquaculture production, and have high economic and societal value. They, however, show often enigmatic differences in their physiology (27), especially the responsiveness and susceptibility to stress (28) and react differently to an acute stressor, when previously exposed to chronic (crowding) stress (29–33). Moreover, seabream seems more resilient than seabass in terms of growth under stress (31, 32, 34). Based on that and to study the effects of different stress loads on the response and identify key neuroendocrine features that regulate these differences between these species, seabass and seabream were exposed to increasing levels of repeated stress episodes combining common aquaculture stressors, such as confinement, chasing and air-exposure (as a model for chronic stress) for 3 weeks and were then subjected to an acute stress test [ACT; (35)]. Fish were sampled for “baseline values” and 1-h post-stress to assess interrenal steroid production capacity. The general performance of fish (food intake and growth) was monitored over the experiment; levels of plasma cortisol and α-MSH were quantified at the end of the experiment. *In-situ* hybridization of the immediate early gene *cfos* was carried out to give anatomical resolution in gene activity; then expression of a set of key target genes in the telencephalon and preoptic area was analyzed.

## Materials and methods

### Animals

Hatchery produced seabass (14-months-old) and seabream (12-months-old) were provided by the Institute of Marine Biology, Biotechnology and Aquaculture of the Hellenic Centre from Marine Research (HCMR) and Forkys S.A. (Sitia, Greece), respectively. In total 160 seabass of 28.69 ± 4.04 cm (mean ± SD) fork length and 380 ± 83.1 g body mass and 160 seabream with 25.05 ± 1.14 cm fork length and 322 ± 54.8 g body mass were used. Fish were kept at HCMR in Gournes, Crete, Greece. Duplicate groups of fish were divided according to body weight over eight cylindrical 500-L tanks with flow-through filtered seawater at a final stocking density of 16.2 ± 0.2 kg m^−3^ for seabass and 14.8 ± 0.3 kg m^−3^ for seabream. The fish were then left to acclimatize for 3 weeks before the start of the experiment. The water temperature was kept at 19°C and the photoperiod was set at 12L:12D. Fish were fed *ad libitum* during the experiment and the quantity of the food consumed was measured daily per tank (by collecting uneaten pellets within 1 h after feeding). The feed used consisted of 44% protein and 19% lipids (Irida S.A., Greece).

### Experimental design

The experimental treatment consisted of exposing seabass and seabream groups to three different chronic stress regimes, varying in intensity, over a period of 21 (seabass) or 24 (seabream) days (Table 1). The experiments were conducted in July 2013 for seabass and October 2013 for seabream. The stressors used were chosen in a way that they reflect common aquaculture practices and have been previously shown to elicit stress responses in both species. Specifically, these stressors were confinement (30, 36, 37), confinement and chasing (38, 39) and a combination of confinement, chasing and air-exposure (28, 40) (Table 1). In detail, the low stress regime consisted of subjecting fish to a confinement stressor for 30 min every 2nd day; this was accomplished by lowering a net into the tank to decrease the available space to 50% (doubling the density) while keeping a constant water volume and similar water quality. The medium stress regime consisted of subjecting fish to both confinement (conducted as previously described) and chasing of the fish for 5 min with a net every 2nd day. The high stress regime consisted of confinement (to only 25% of the tank volume) for 30 min, chasing for 5 min every 2nd day, and air exposure for 1 min once per week. These stressors were applied to the fish between 10.00 and 12.30 h.

**Table 1:** Stress applied to seabass and seabream for three different stress loads.

			**Stress load**		
**Stressor**	**Time (min)**	**Frequency**	**Low**	**Medium**	**High**
Confinement^*^	30	Every 2 days	√	√	√
Chasing	5	Every 2 days		√	√
Air exposure	1	Every 7 days			√

*Confinement and chasing were performed once every 2 days; air-exposure was performed once a week*.

* *Confinement in the Low and Medium stress groups was performed by restraining the fish to 50% of the initial water volume, for the High stress group to 25% of the volume*.

Two days after the end of the chronic stress treatments 10 out of 20 fish per tank were immediately sampled (referred to as T0 fish) after netting and deep anesthesia with 0.5% (v/v) 2-phenoxyethanol. Blood was drawn via heparinized syringes, centrifuged (2,000 × *g* for 10 min) and the plasma stored at −80°C until further analysis. The spinal cord was cut to kill the fish and telencephalic, preoptic area and brainstem samples were collected, snap-frozen in liquid N_2_, and stored at −80°C. The 10 remaining fish were acutely stressed by subjecting them to a net chase for 5 min and then air-exposure for 1 min. The fish were then left undisturbed for 1 h [when the peak cortisol response after stress is observed; (28, 37, 40–42)] and deeply anesthetized before sampling (T1 fish), as explained above.

The laboratories of the Hellenic Centre for Marine Research are certified and have obtained the codes for breeding and husbandry of animals for scientific purposes (EL 91-BIO-03, EL 91-BIO-04). All procedures involving the handling and treatment of fish were approved by the HCMR Institutional Animal care and use committee in accordance to Greek (PD 56/2013) and EU (Directive 63/2010) legislation on the care and use of experimental animals following the principles of refinement, replacement and reduction in animal experimentation.

### Plasma analysis

Plasma cortisol levels were determined by radioimmunoassay, according to Gorissen et al. (43). Plasma α-MSH levels were evaluated by radioimmunoassay using the L9 α-MSH antibody (44). The antiserum shows 100% cross-reactivity with des-, mono-, and di-acetyl α-MSH. Tracer α-MSH-peptide was labeled with ^125^I through the iodogen method (45).

### Brainstem 5-HT neurochemistry

Frozen brain stems were homogenized in 4% (w/v) ice-cold perchloric acid (PCA) containing 0.2% EDTA and 40 ng ml^−1^ epinine (deoxyepinephrine as an internal standard) with a Potter–Elvehjem homogenizer. After centrifuging samples for 5 min at 15,493 rcf, the supernatant was analyzed by high-performance liquid chromatography (HPLC). The mobile phase was; 12 μmol L^−1^ EDTA, 86 mmol L^−1^ sodium phosphate and 1.4 mmol L^−1^ sodium octyl sulfate in deionized water (resistance 18.2 MΩ cm^−1^), containing 7% acetonitrile; pH was set to 3.1 with phosphoric acid. The system consisted of a solvent delivery system (Shimadzu, LC-10AD, Kyoto, Japan), an auto-injector (Famos, Spark), a reverse phase column (4.6 × 100 mm, H0ichrom, C18, 3.5 mm) and an ESA Coulochem II detector (ESA, Bedford, MA, USA) with two electrodes at −40 and +320 mV. A conditioning electrode with a potential of +40 mV was used to oxidize possible contaminants before analysis. Brain stem concentrations of 5-HT and the 5-HT metabolite 5-Hydroxyindoleacetic acid (5-HIAA) were quantified by comparison with standard solutions of known concentrations and corrected for recovery of the internal standard using HPLC software (CSW, DataApex Ltd, Prague, the Czech Republic). The 5-HT turnover was quantified by ratio of 5-HIAA/5-HT.

### RNA isolation

Brain tissue was dissected into telencephalon and preoptic area using a stereo microscope, as *per* Madaro et al. (35, 46). Tissues were homogenized in TRIzol reagent (Gibco BRL) according to manufacturer's instructions. RNA concentration and purity were determined by measuring absorbance at 260 and 280 nm with Nanodrop® ND-1000 UV–Vis spectrophotometry (Peqlab, Erlangen, Germany).

### Synthesis of cDNA

Synthesis of cDNA was performed as *per* Madaro et al. (35, 46). RNA (100–500 ng) was reverse-transcribed by a series of incubations: 10 min at 25°C, followed by 50 min at 42°C and 15 min at 70°C; cDNAs were then diluted five times and stored at −20°C until further analysis.

### Real-time quantitative PCR

Oligonucleotides used in the qPCR analysis are shown in Table 2. To each diluted cDNA sample, 16 μl of a mix containing: 10 μl iQ™ SYBR® Green Supermix (2x) (Bio-Rad, Hercules, CA, USA), 0.7 μl (10 μM) primer forward, 0.7 μl (10 μM) primer reverse, 4.6 μl DEPC H2O was added. The amplification protocol was carried out on a CFX96 Touch™ Real-Time PCR Detection System (Bio-Rad, Hercules, CA, USA) and consisted of 3 min at 95°C, followed by 40 cycles of amplification (95°C for 15 s and 60°C for 1 min). A melting curve was generated for each sample to assess specificity of the PCR products.

**Table 2:** Primer sequences used in RT-qPCR for seabass and seabream.

	**Gene**	**Forward primer 5^′^to 3^′^**	**Reverse primer 5^′^to 3^′^**	**Accession no**
Seabass (*D. labrax*)	*crf*	CGCTACGAATGTCGGGCTAT	GGGAGTTTTGGGTTTGGGGA	JF274994
	*gr1*	TCAGTGGCTTGCTCAAGGAG	GGGCTTCTGCTGGTGAGAAT	AY549305
	*mr*	CCTGTCTCCTCTATGAATGG	AATCTGGTAATGGAATGAATGTC	JF824641
	*elf1α*	CAAGGAGGGCAATGCCAGT	GAGCGAAGGTGACGACCAT	AJ866727
	*rpl17*	TTGAAGACAACGCAGGAGTCA	CAGCGCATTCTTTTGCCACT	AF139590
	*pomca*	CAGAGACACCGATCATCCCG	TCTTCAGGGAAAACCTCGGC	AY691808
Seabream (*S. aurata*)	*crf*	CGCTACGAATGTCGGGCTAT	GGGAGTTTTGGGTTTGGGGA	KC195964
	*crf-bp*	GATTTCGTGCAGCTGTTGGG	CAGCCGATCTTCATGTGGGT	KC195965
	*gr1*	AGTGCTCCTGGCTCTTCCTA	GCTTCATCCGCTCCTCGTT	DQ486890
	*mr*	CGCCTGGCTGGAAAGCAGATG	GAGGTCAGGGGCAAAGTAGAGCAT	(47)
	*elf1α*	TGGTGATGCTGCCATTGTC	AGCCACTGTCTGCCTCAT	AF184170
	*fau*	AGCCCAACTCTGCCATCA	AATCCTGCCACCAGAACCT	(47)
	*pomca1*	CCGCTGCTCACGCTCTTC	GGCTGCTCGTCTTCTGTCTCT	(47)

*The sequence for seabass crf-bp was not available at the time of experimentation*.

### *In-situ* hybridization

For *in-situ* hybridization fish were sampled directly from their holding tank (at basal conditions, *n* = 2/species) and 1 h post-stress conditions (chasing for 5 min and air exposure for 1 min, *n* = 2/species). All fish were quickly and deeply anesthetized with 1% (v/v) phenoxyethanol and fixed by vascular perfusion with 4% PF in 0.1 M Sørensen's phosphate buffer (PB; 28 mM NaH_2_PO_4_, 71 mM Na_2_HPO_4_, pH 7.2). Dissected brains were post-fixed in the same fixative for 16 h at 4°C. The tissue was washed three times 20 min in PB, cryopreserved overnight in 25% sucrose in PB at 4°C, embedded in Tissue-Tek OCT-Compound (Sakura Fintek) and stored at −80°C until sectioning.

Adjacent transverse 12 μm sections were cut with a Leica CM 1850 cryostat (Leica Microsystems, Wetzlar, Germany), collected on SuperFrost Ultra Plus glasses (Menzel Glaser, Braunschweig, Germany) and dried at 65°C for 10 min. Digoxigenin-labeled riboprobes were prepared with a digoxigenin (DIG)-RNA labeling mix following the manufacturer's instructions (Roche Diagnostics, Mannheim, Germany). The *cfos* ISH probes for seabream and seabass were 542 and 467 nucleotides long, respectively. Forward GGCTCGAGTTCATTCTCGCT and reverse GTCGTTGCTGTTGCTTCCTC and forward TCTGGGATGGTGGTCTGTGA and reverse CCAGCCTTTGATCTCCTCGG primers were used to clone the *cfos* probe primers in seabream and seabass, respectively. The quality and quantity of the synthesized riboprobes were assessed by agarose gel electrophoresis. Pretreatment and treatment of sample for ISH was conducted as specified earlier (48). The reaction with chromogen substrate (3.4 μl of nitroblue-tetrazolium, 3.5 μl of 5-bromo-4-chloro-3-indoylphosphate (Roche Diagnostics, Indianapolis, IN, USA) and 0.24 mg/ml levamisole in visualization buffer) was carried out for 3–24 h in darkness at room temperature (samples were routinely checked to avoid overstaining). The reaction was terminated with stop solution (10 mM Tris-HCl, 1 mM EDTA, 150 mM NaCl, pH 8.0) and tissue was mounted in ProLong Gold (Invitrogen, Carlsbad, CA, USA). Photomicrographs were taken by a digital camera (Leica DFC 320, Leica 350 FX) attached to a Leica DM 6000B microscope using the LEICA APPLICATION SUITE, version 3.0.0 image acquisition and processing software.

### Statistical analysis

For plasma analyses and gene expression data, normal distribution of data was tested with the D'Agostino and Pearson omnibus normality test. Cortisol data were analyzed using linear regression. Other plasma analyses were assessed by two-way ANOVA, gene expression data and brain mono-amine data were tested using one-way ANOVA. Significance of effects were subsequently determined by Tukey's *post-hoc* tests or unpaired Student's *t*-testing, where appropriate (α-level was adjusted for multiple comparisons). For all statistical tests *P* < 0.05 was taken as the fiducial limit, unless otherwise stated (in case of multiple comparisons). All statistical analyses were performed with GraphPad Prism 7.0 (GraphPad Software Inc., La Jolla, CA, USA).

## Results

### Chronic stress, growth, and food intake in seabass and seabream

**Figure 1:**
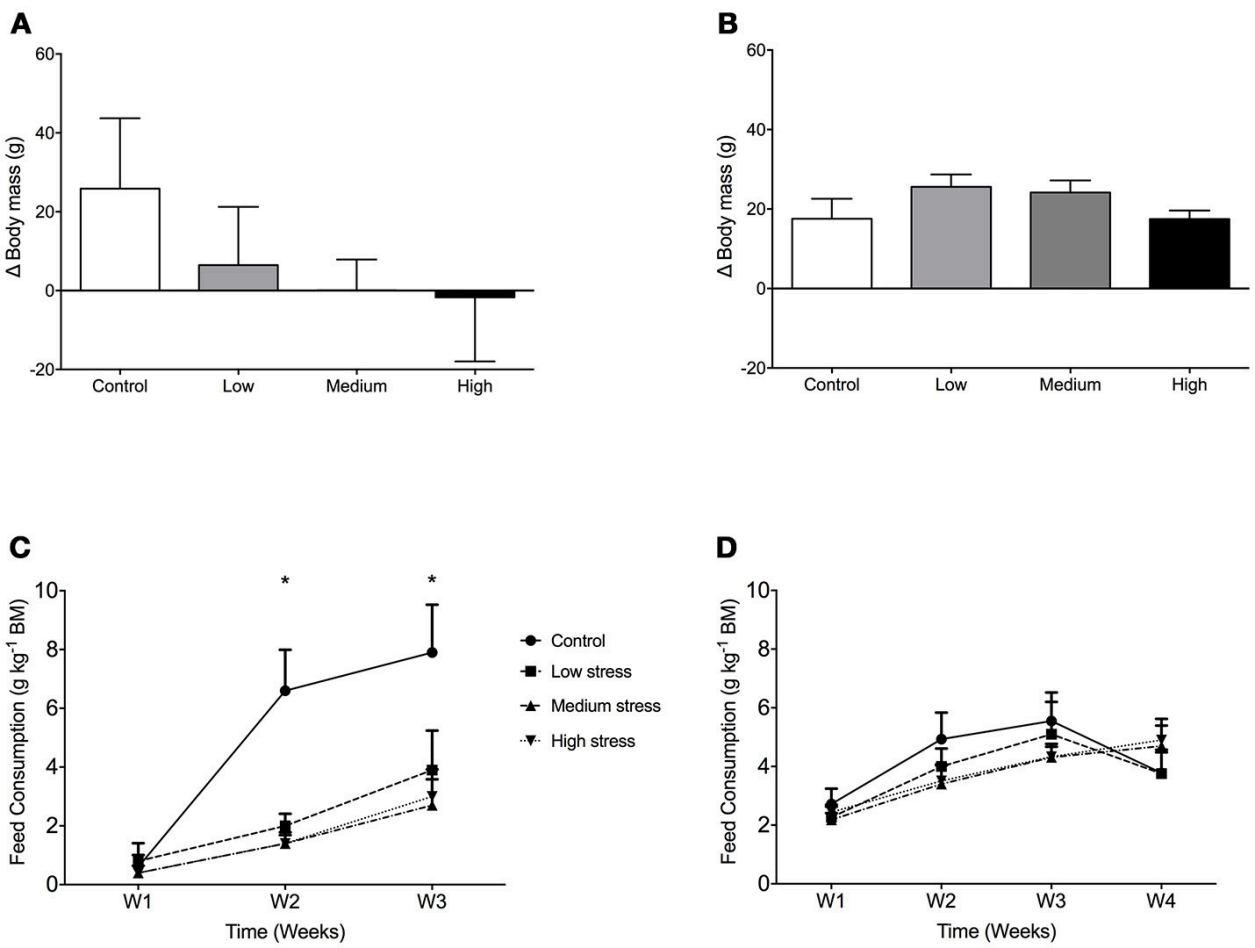
Growth and daily food consumption in sea bass and seabream. Body mass gain in seabass **(A)** and seabream **(B)** in controls and after exposure to low, medium or high chronic stress (mean + 1 SD; *N* = 2; *n* = 40). Daily food consumption of seabass **(C)** and seabream **(D)**, expressed as gram dry food per kg of fish. Two-way ANOVA showed significant differences between control and the rest of the groups in seabass (^*^P < 0.05).

In seabass growth decreased with increasing stress intensity, not in seabream (Figures 1A,B). For feed consumption, there was a significant interaction for seabass between stress and time [*F*_(6, 95)_ = 2.36; *P* = 0.037], with higher feed consumption in controls compared to stressed groups in the 2nd and 3rd week of the experiment (Figure 1C). In seabream, no differences in feed consumption were observed among any of the groups [*F*_(3, 143)_ = 0.45; *P* = 0.717] (Figure 1D).

### Plasma cortisol, stress load and acute stress response

**Figure 2:**
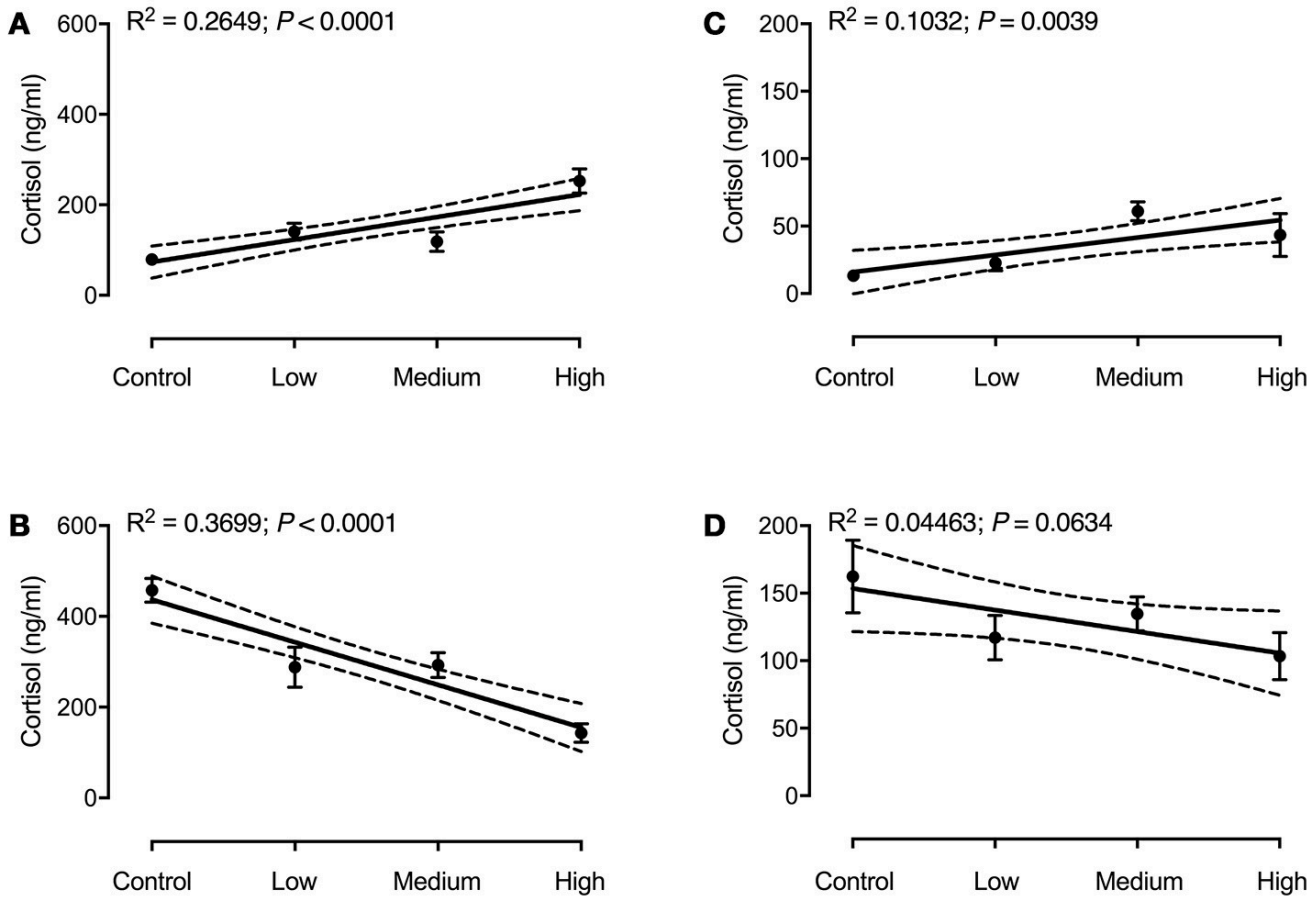
Plasma cortisol in seabass **(A,B)** and seabream **(C,D)** in controls and after exposure to chronic stress, before **(A,C)** and after an acute stress test **(B,D)**. Data are expressed as mean ± SEM (*N* = 2; *n* = 20). Linear regression (solid line) results are shown in each panel. The 95% confidence interval is shown as dashed lines.

Regression analysis of seabass plasma cortisol showed a significant effect of stress load on basal cortisol levels [*F*_(1, 75)_ = 27.03; *P* < 0.0001; *R*^2^ = 0.2649; Figure 2A], as well as a significant effect of the ACT [*F*_(1, 76)_ = 44.61; *P* < 0.0001; *R*^2^ = 0.3699; Figure 2B]. Basal cortisol levels increased with increasing stress load, whereas plasma cortisol after the ACT decreased with increasing stress load. For seabream a significant regression between stress load and plasma cortisol was found for basal cortisol only [*F*_(1, 77)_ = 8.86; *P* = 0.0039; *R*^2^ = 0.1032; Figure 2C], not for plasma cortisol after the ACT [*F*_(1, 76)_ = 3.55; *P* = 0.0634; *R*^2^ = 0.04463; Figure 2D]. There were significant interactions between chronic and acute stress in both species [*F*_(3, 147)_ = 29.27; *P* < 0.0001 for seabass, and *F*_(3, 149)_ = 3.37; *P* = 0.0178 for seabream].

### Plasma α-MSH levels and chronic stress

In both species no effect of chronic stress treatments on basal plasma α-MSH was observed (data not shown), nor was any interaction effect found between chronic and acute stressors. Values varied around 270 pM for seabass and 250 pM for seabream.

### Monoamines in the brain

In both species no effect of chronic stress on brain stem monoamine content was observed (data not shown). 5-HT turnover (as quantified by 5-HIAA/5HT ratio's) ranged between 0.40 and 0.50 for seabream and 0.25 and 0.30 for seabass.

### *In-situ* hybridization of *cfos*

**Figure 3:**
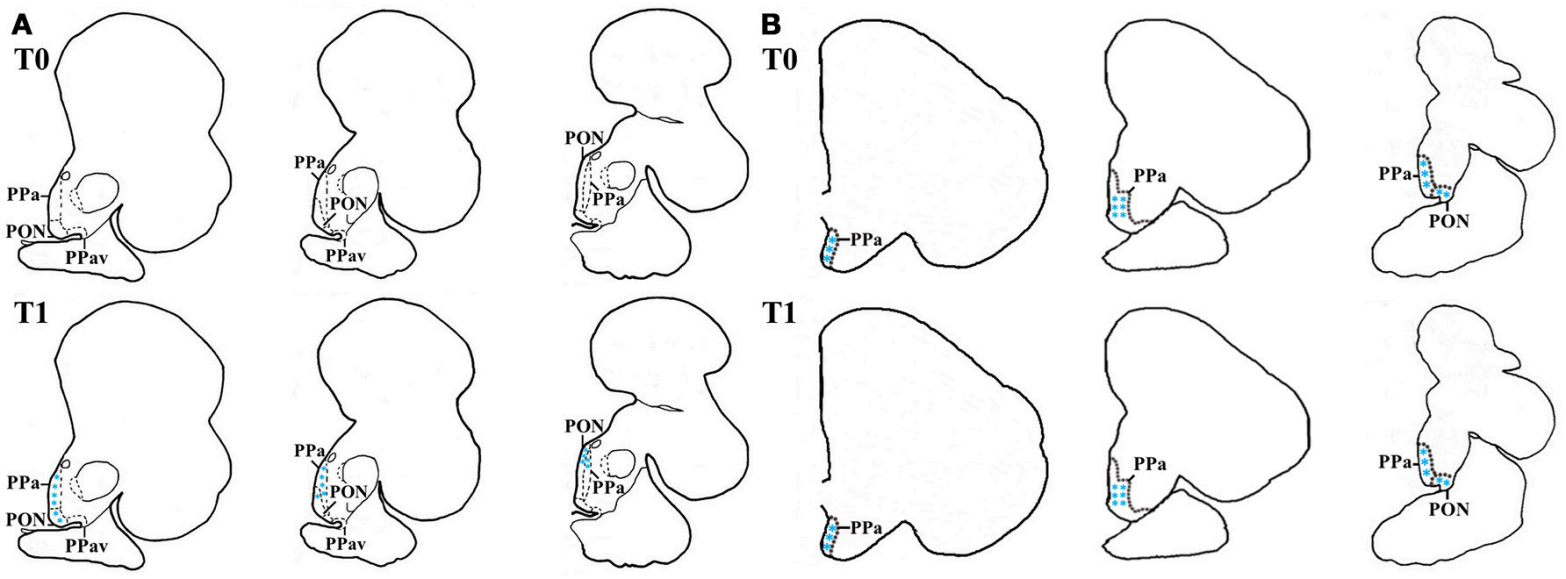
*In-situ* hybridization of *cfos* in seabass and seabream brain before (T0) and after acute stress (T1). Schematic representation of transverse brain sections containing the POA in seabream **(A)** and seabass **(B)** illustrating *cfos* mRNA transcript abundance before (T0) and after acute stress (T1). The blue stars represent labeled cells within each area.

There were species-specific differences in the *cfos* mRNA abundance in the preoptic area, particularly at basal levels. That is, while no labeling of *cfos* mRNA was seen in seabream samples, in seabass high mRNA abundance was found in the preoptic area. This suggests activation of the POA at basal conditions in dependence of degree of stress load. Notably, *cfos* abundance increased in seabream and remained high in seabass post-stress (Figures 3A,B).

### Gene expression in POA

**Figure 4:**
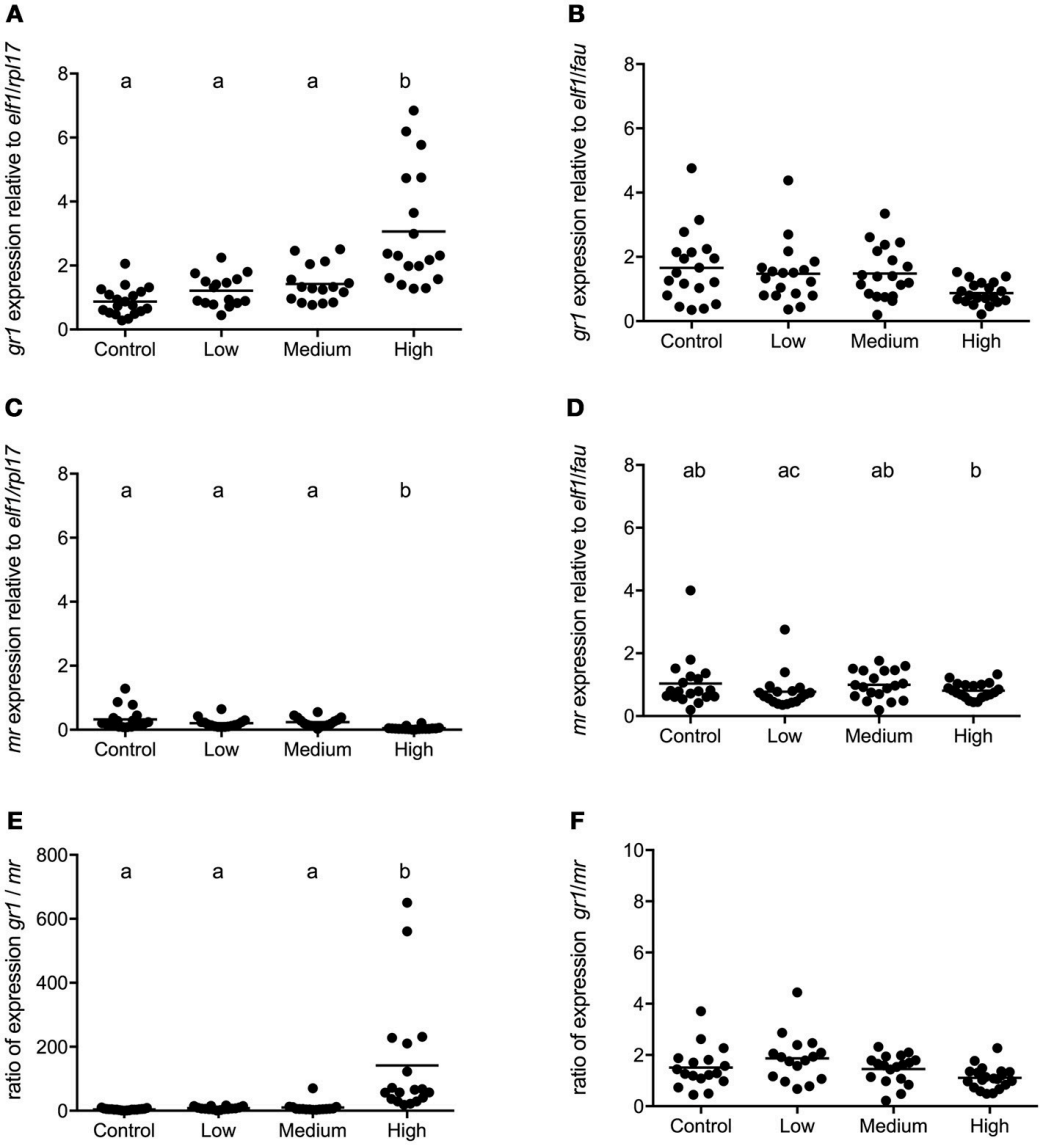
*gr1* and *mr* gene expression in POA of seabass and seabream. Expression of *gr1, mr* and *gr1*/*mr* ratio in seabass **(A,C,E)** and in seabream **(B,D,F)** for control fish and for groups previously subjected to chronic stress. Data are shown for individual fish; the black lines indicate the mean (*N* = 2; *n* = 20). One-way ANOVA showed a significant effect of chronic stress; different letters indicate significant differences between groups (*P* < 0.05).

In seabass, the *gr1* and *mr* expressions had increased and decreased, respectively, in the high stress group compared to all other groups [*gr1*: *F*_(3, 68)_ = 16.50; *P* < 0.0001, *mr*: *F*_(3, 68)_ = 25.94; *P* < 0.0001; Figures 4A,C]. Consequently, the *gr1*/*mr* ratio was significantly higher in the high stress group compared to all other groups [*F*_(3, 68)_ = 47.60; *P* < 0.0001; Figure 4E]. In seabream no significant differences were found in the expression of *gr1* and *mr* (Figures 4B,D) or in the *gr1/mr* ratios (Figure 4F).

**Figure 5:**
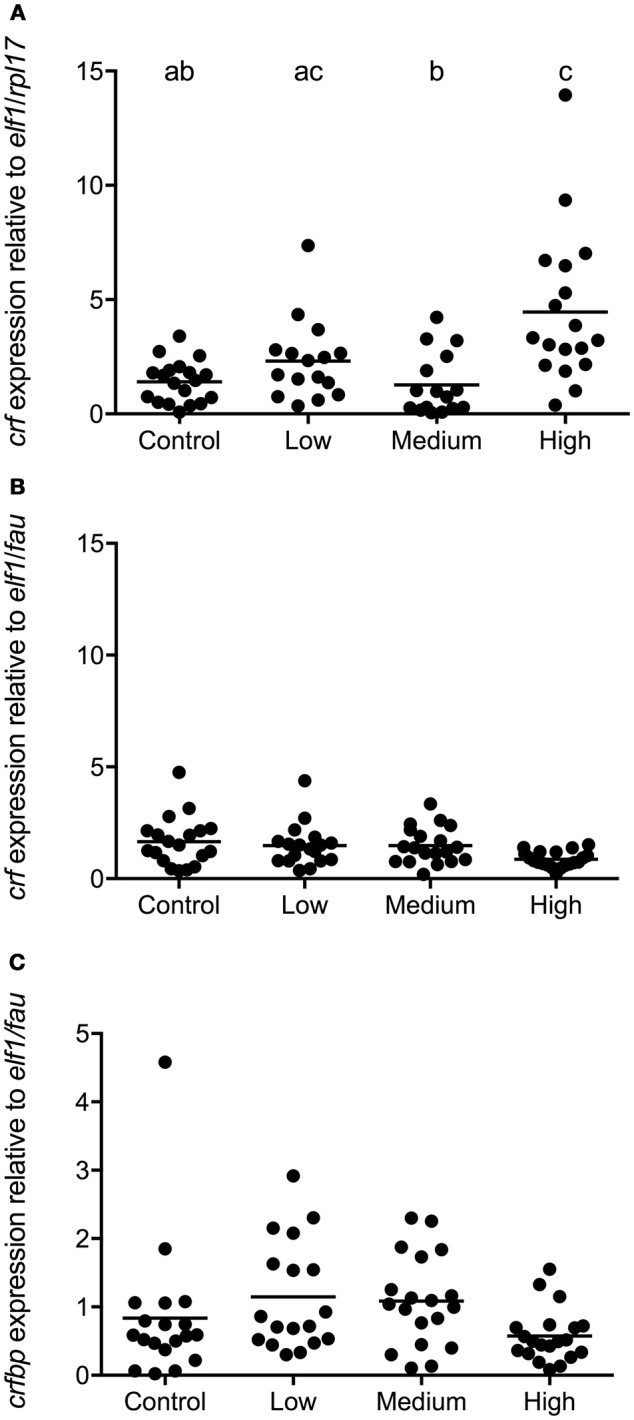
*crf* and *crf-bp* gene expression in POA of seabass and seabream. Expression of *crf* in seabass **(A)** and in seabream **(B)**, as well as *crf-bp* in seabream **(C)**, for control fish and for groups previously subjected to chronic stress. Data are shown for individual fish; the black lines indicate the mean (*N* = 2; *n* = 20). One-way ANOVA showed a significant effect of chronic stress; different letters indicate significant differences between groups (*P* < 0.05).

In seabass POA *crf* expression was affected by the intensity of chronic stress [*F*_(3, 68)_ = 8.974; *P* < 0.0001]. In this species the expression of *crf* was higher in the high stress compared to the control and medium stress groups (Figure 5A). In seabream, no significant differences in *crf* and *crf-bp* expression were evident between groups (Figures 5B,C). No primer sequence for *crf-bp* in seabass was available at the time of these studies.

A significant correlation between *gr1* and *crf* (Spearman *r* = 0.570; *P* < 0.0001) was found for seabass, for all experimental groups. For seabream there was no significant correlation between these parameters (Spearman *r* = −0.1076; *P* = 0.379).

### Gene expression in pituitary gland

**Figure 6:**
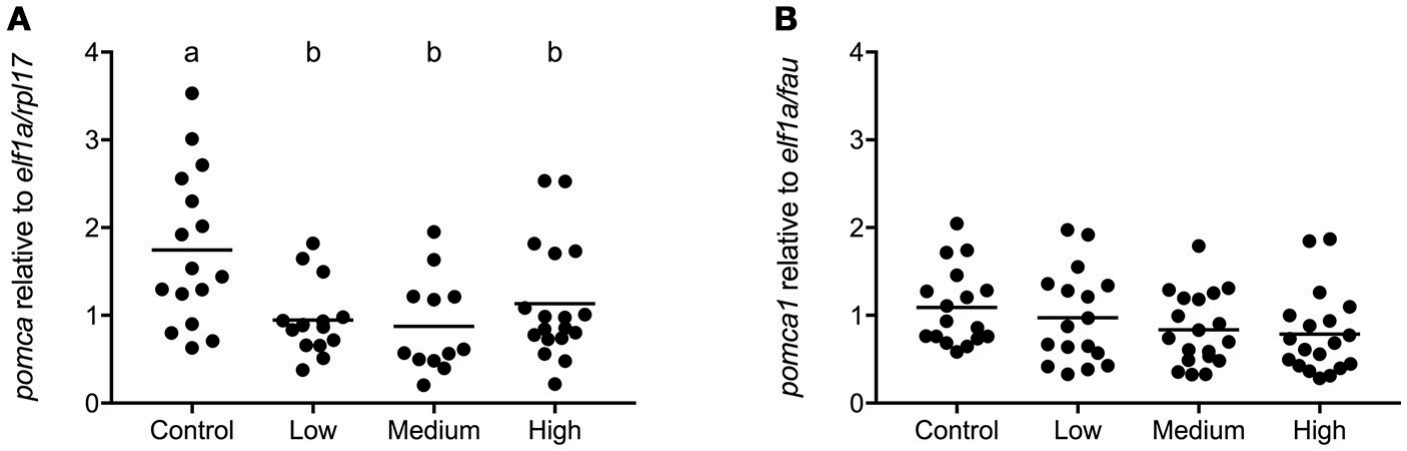
*pomca* gene expression in pituitary gland of seabass and seabream. Expression of *pomca* in seabass **(A)** and in seabream **(B)**, for control fish and for groups previously subjected to chronic stress. Data are shown for individual fish; the black lines indicate the mean (*N* = 2; *n* = 20). One-way ANOVA showed a significant effect of chronic stress in seabass only; different letters indicate significant differences between groups (*P* < 0.05).

In seabass low, medium, and high levels of chronic stress decreased transcript abundance of *pomca* [*F*_(3, 57)_ = 5.434; *P* = 0.002; Figure 6A]. In seabream no significant effect of chronic stress on *pomca1* expression was observed [*F*_(3, 68)_ = 1.574; *P* = 0.20; Figure 6B].

### Gene expression in telencephalon

**Figure 7:**
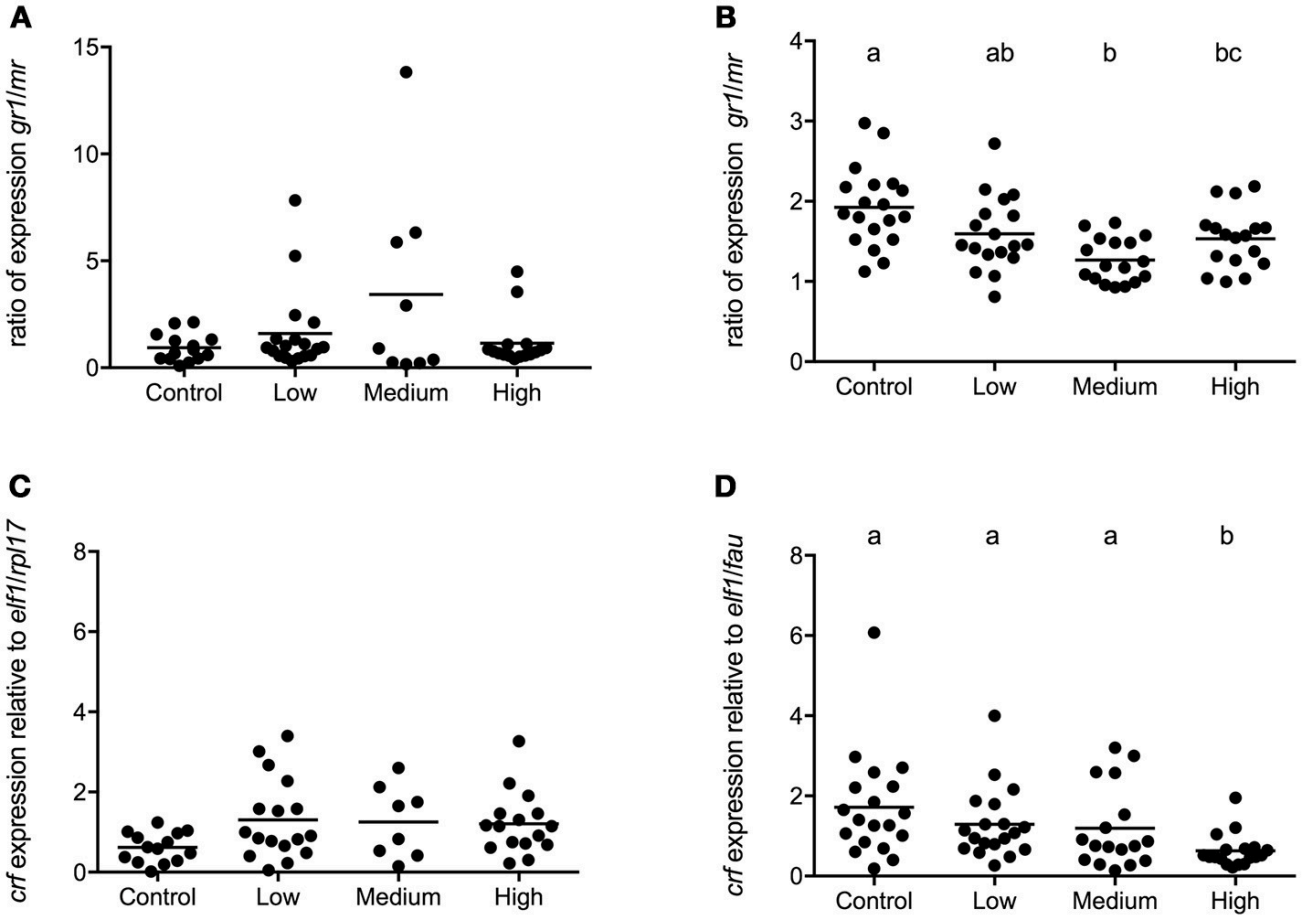
Gene expression in telencephalon of seabass and seabream. Expression of *gr1*/*mr* **(A)** and *crf* **(C)** in seabass and *gr1*/*mr* **(B)** and *crf* **(D)** in seabream for controls and for groups previously subjected to chronic- low, medium, and high stress. Data are shown for individual fish; the black lines indicate the mean (*N* = 2; *n* = 20). One-way ANOVA showed a significant effect of chronic stress; different letters indicate significant differences between groups (*P* < 0.05).

In seabass a high degree of variation in telencephalic gene expression was observed and chronic stress further increased this variation. No statistical differences existed in the expression of *crf* and unlike the pattern in POA, the *gr1*/*mr* ratio was not affected by chronic stress load (Figures 7A,C).

In seabream, telencephalic *crf* mRNA levels were significantly lower in the highly stressed seabream compared to all others [*F*_(3, 72)_ = 5.03; *P* = 0.0033] while the *gr1*/*mr* ratio had decreased as stress load increased (Figures 7B,D).

## Discussion

Insight in fish stress handling is crucial to guarantee welfare and product quality in aquaculture and fisheries (49). In the present study, we compared two fish species with great relevance to Mediterranean aquaculture that differ widely in their life history and stress handling capacities.

It is well known that stress is energy consuming, leads to decreased food consumption and thus growth in fish (50–52). Indeed, the seabass decreased their feed consumption due to chronic stress. Moreover, body mass decreased with increasing stress load. Both feed consumption and growth were unaffected by a similar stress imposed on seabream. From these observations, we conclude that the stress load in this study was significant but not extreme and that seabream apparently are more resilient.

In general, it is believed that reduced feeding intake induced by stress is regulated by a combination of behavioral and physiological adaptations to stress (53). These adaptations alter energy expenditure allocation (50, 51), which may in turn lead to growth reduction. Seabass individuals are sensitive to common aquaculture practices such as tank cleaning, which can lead to reduced feed intake for up to 3 days (54), and a significant reduction in growth (51, 55). On the contrary, seabream seems to be more resilient to stress, and did not show differences in growth between control and daily-stressed fish (32). Taken together our data confirm earlier reports showing lower resilience of seabass to stress compared to seabream.

Cortisol in fish combines glucocorticoid and mineralocorticoid actions, by redistributing energy away from growth and reproduction toward survival mechanisms including regulation of hydromineral balance (8, 56, 57). Therefore, high and persistent elevated concentrations of circulating cortisol can affect a wide range of metabolic, immune and reproductive functions (8, 25).

It is shown here that seabass subjected to increasing intensity of (chronic) stress mildly elevate basal plasma cortisol levels (range: 50–200 ng/ml; Figure 2) compared to controls; remarkably, basal levels of cortisol in seabass are remarkably high compared to the generally accepted “non-stress” level seen in most fish (up to 20 ng/ml). Seabass is in general characterized by high cortisol values and variation (27, 28, 39, 58, 59), and the current results point out that chronic stress can further increase these high (basal) cortisol levels.

The decreasing response in seabass to the ACT with increasing stress load history indicates that cortisol production capacity is impeded when the stressor persists, the interrenal tissue becomes exhausted (46, 60, 61). In other words, the stress intensity in severely stressed fish exceeded their coping ability (62, 63), the stress given presented an allostatic overload (3). Indeed, repetitive common handling stress on this species, such as tank cleaning (51) or exposure to high-density stress (30, 31) cause changes in circulating cortisol levels. It seems therefore that the intensity and type of the (chronic) stressor and the sum of stressors imposed (e.g., handling, suboptimal water quality and light conditions) need consideration in defining their effects on cortisol response and stress regulation in seabass. In this respect, small-scale laboratory experiments such as presented here are highly informative in aquaculture policy making.

Contrary to what was observed in seabass, in seabream no significant differences were observed in cortisol levels between chronically stressed groups at basal conditions, and all groups responded with increased cortisol to acute stress, and we take this to indicate a healthy physiological functioning of the HPI axis in this species and strong capability to handle stress. The outcome of this comparative study makes us confident that the stress imposed reflects (presumed) realistic conditions.

In our studies we did not bisect the pituitary gland into pars distalis and pars intermedia, we did not isolate ACTH- or MSH-cells, and therefore *pomc* expression levels shown could reflect both ACTH and MSH activities. Only, in seabass we found an inhibitory effect of stress on *pomc* expression, while preoptic *crf* expression was unaffected (low and medium stress) or up-regulated (high stress); so *pomc* expression had increased either to replenish POMC-derived protein stores or an as yet unknown short feedback loop affects the pituitary gland under stress in this fish.

Plasma α-MSH in some species may serve as modulator of the stress response (40, 64), and particularly under chronic stress conditions α-MSH may act as (mild) corticotrope (8, 18), lipolytic, or anorexigenic signal (3). At present little is known about *plasma* α-MSH actions on brain functioning in relation to feeding; The option of plasma MSH as signal to brain (stem) centers [α-MSH is a cyclic molecule which may easily and passively pass the blood brain barrier; (65) involved in feeding control requires further studies. It has been reported for Mozambique tilapia (*Oreochromis mossambicus*) that plasma α-MSH is only regulated under chronic stress conditions, but not after an acute stressor (66). In their studies on seabream, Arends and colleagues air-exposed naïve fish for 3 min and reported a very high peak in cortisol level (1,400 vs. 414 nM in this study after an ACT). These high cortisol levels correlated with elevated MSH-levels (which we did not observe in the present study) from which then was concluded that air-exposure has a major effect on catecholaminergic pathways as ACTH was not into play (40). Major differences in experimental design (e.g., 1 vs. 3 min air-exposure, chasing before air-exposure, pre-conditioning to different stress levels) may make the difference in outcome between these two studies. Importantly, habituation of the catecholaminergic response induced by the chronic stress application cannot be excluded. Indeed, in our experiment fish responded to acute stress with an increase in plasma cortisol, not in α-MSH levels. Possibly, acetylation of α-MSH (independent from total levels of α-MSH) is affected by chronic stress. The corticotropic activity of α-MSH in Mozambique tilapia increases with increasing degree of acetylation (des-, mono- di-acetyl α-MSH) (18) and a shift in α-MSH species (apart from total levels) could result in a differential contribution of α-MSH to cortisol production. However, these aspects were not analyzed in the present research. The consequence of acetylation of the POMC-derived peptides MSH and endorphin(s) is differential: MSH may become more biopotent, endorphins become inactivated by acetylation (18, 66). Is it the protection against the powerful actions of endorphins to consider in POMC-peptide stress regulation? More detailed studies are needed.

No differences in the 5-HT turnover rate were observed between chronic stress groups in both species. Generally, mammalian studies show that chronic stress and increased allostatic load affect 5-HT neurochemistry [reviewed by Beauchaine et al. (67). Similarly, chronic stress, induced by high stocking densities, resulted in elevated basal levels of brain stem 5-HT turnover in rainbow trout (68). However, upon an acute stress, already chronically stressed trout showed blunted stress responses including telencephalic 5-HT responsiveness (69). However, the present results indicate that chronic stress does not affect basal 5-HT neurochemistry, which is somewhat in contrast to the aforementioned rainbow trout studies. However, it is important to point out that in the rainbow trout studies fish were exposed to a continuous stressor, while in the present study they were repeatedly exposed to a combination of high-intensity stressors (different densities, chasing and air-exposure). Of note, the experimental design of the present study did not include brain 5-HT responsiveness to an acute stressor.

Stress can significantly alter the expression profile of genes related to metabolic, immune and cell signaling functions (70–72). The expression of glucocorticoid receptors and heat shock proteins is altered when seabass are chronically stressed by high rearing density (71, 73). Changes in the expression of stress-related genes have also been reported in seabream exposed to different rearing densities (33) or to unpredictable chronic low intensity stress in the early stages of life (74).

In the present study there was a remarkable difference in basal *cfos* expression in POA of seabass and seabream. In seabream the gene was apparently and essentially silent in unstressed seabream, but *cfos* expression was clearly seen after acute stress. In seabass, *cfos* expression in the POA was found under basal as well as post-stress conditions. It has been suggested that *cfos* expression is up-regulated after acute exposure to (hypercapnia) stress in seabass (72). Moreover, in zebrafish *cfos* expression seems to be upregulated after exposure to chronic stress (75). Still, however, literature on this aspect is limited and we can only speculate that the high expression of *cfos* confirms and reflects high HPI-axis activity in seabass, in agreement with the endocrine pre- and post-acute stress concentrations of cortisol, glucose and lactate in seabass, compared to those of seabream (28).

The significant increase in POA *crf* expression in the highly stressed seabass indicates that their impaired cortisol response to acute stress is not related to a dysfunction of the POA, but must be sought rather in exhaustion of the interrenal tissue (as discussed above) or in the pituitary corticotropes (35). Indeed, the seabass interrenal gland appears to be the key tissue where regulation of cortisol responsiveness occurs (Samaras and Pavlidis, submitted). Meanwhile, seabream coped well with the stress imposed. These fish presented both low cortisol levels and unaltered *crf* expression in the POA. This is in agreement with results reported for Atlantic salmon subjected to a similar unpredictable chronic stress (35). Taken together, the present study shows a species-specific regulation of the HPI axis to chronic stress.

A profound difference in cortisol receptor profile was found between seabass and seabream. The *gr1*/*mr* ratio showed an over 100-fold increase in highly stressed seabass, compared to control groups, while in seabream the ratio remained unaffected by stress. The drastic ratio shift in seabass resulted from a combined increase in *gr1* expression and decrease in *mr-*expression; we speculate that this shift is best explained by differential feedforward and feedback mechanisms of cortisol on these targets, respectively. Shifts in *gr*/*mr* ratio are indicators of impaired appraisal, poor learning and fear avoidance in vertebrates (76–78). In zebrafish (79) and trout (80) chronic stress increased the brain *gr*/*mr* ratio and this was associated with diminished cognitive quality and inhibitory avoidance learning. In mammals, *gr*/*mr* ratio shifts make the brain prone to steroid-induced pathologies (81) and we suggest here that the same may hold for fish (82–84).

If we take the *gr/mr* ratio as indicator of allostatic load [as done in rodent studies; (77, 81, 85)], also in fish, then our chronic stress paradigm induces allostatic overload and thus the ratio may be considered an appropriate indicator of stress load. We propose that such a receptor profile is a trait common to vertebrates, and originally developed in fish, the earliest vertebrates.

Finally, the telencephalon is an important target for cortisol feedback, illustrated by changes in *gr/mr* ratio in e.g., zebrafish (82–84). Indeed, in seabream we observed both decreasing *gr*/*mr* ratio's and *crf* expression levels with increasing stress load. To appreciate a stress response it is important to recognize and appreciate the role of complex behavior in this response, memory, learning, appraisal and prediction are crucial in coping with a dynamic environment and requires brain structures that facilitate such behavior. Evidence is accruing that the fish telencephalon/forebrain contains structures homologous and partly analogous to the mammalian hippocampus, amygdala, pyriform cortex, and isocortex (3). For zebrafish we have shown via inhibitory fear avoidance learning that the amygdala equivalent (dorsomedial pallium) is involved in acquisition of memory, a likely process involving MR activity, while in hippocampal neuronal clusters (dorsolateral pallium) GR facilitates consolidation of memory (86). A surprising functional parallel seems to exist in fish and mammalian system (81) steering stress-related behavior. The absence of this response in *gr*/*mr* ratio's and *crf* expression in seabass to chronic stress corroborates the notion that this species resides outside its allostatic comfort zone in the current experimental paradigm.

## Conclusions

In this experiment seabass and seabream were found to react very differently to stress. Specifically, seabass appear to be more susceptible to stress in terms of reduced food intake and growth, as well as the regulation of plasma cortisol levels. Seabream compared to seabass appeared to have a strong resistance and lower sensibility to the stress regimes used in this experiment. This study substantiates species-specific differences in (endocrine and neuroendocrine) stress physiology from gene expression to growth performance and (learning) behavior. These considerations on species-specificity should draw attention of those involved in diversification programmes in aquaculture practices.

## Author contributions

AS, NP, MP, LE, GF, and MG conceived and designed the experiments. AS, NP, NM, FS, LE, GF, and MG carried out the experiments. CE, EH, TP, JZ, and MV analyzed the samples. AS, CE, GF, and MG analyzed, interpreted the data, and drafted the manuscript. All authors have critically revised and approved the manuscript.

### Conflict of interest statement

The authors declare that the research was conducted in the absence of any commercial or financial relationships that could be construed as a potential conflict of interest.

## Abbreviations

5-HIAA: 5-hydroxyindoleacetic acid
5-HT: 5-hydroxytryptamin
α-MSH: *alpha-*melanocyte-stimulation hormone
*crf*: corticotropin-releasing factor
*crf-bp*: corticotropin-releasing factor binding protein
*gr1*: glucocorticoid receptor 1
HPI axis: Hypothalamus–Pituitary–Interrenal axis
*mc2r*: melanocortin receptor type 2
*mr*: mineralocorticoid receptor
MRAPs: melanocortin receptor associated proteins
POA: preoptic area
ACT: acute stress test.
